# Supplementary material for: A Rapid and Simple Assay Correlates In Vitro NetB Activity with Clostridium perfringens Pathogenicity in Chickens
Source: Microorganisms. 2021 Aug 11;9(8):1708. doi: 10.3390/microorganisms9081708 (PMC8400579; doi:10.3390/microorganisms9081708)
Supplement: Supplementary file 1 [file microorganisms-09-01708-s001.zip › Document S1_Rabbit immunization protocol.pdf]

Development of polyclonal antiserum towards non-NetB *C. perfringens* proteins: Rabbit immunization protocol

Development of polyclonal antiserum towards the non-NetB *C. perfringens* haemolysis should be performed using a **NetB-negative *C. perfringens* type A strain** with proven ability to produce alpha toxin and perfringolysin O. Native toxins (*C. perfringens* culture supernatants) should be used to the immunize the rabbits. Formaldehyde-inactivation of the culture supernatants results in the development of anti-alpha toxin antibodies which are not able to neutralize the activity of this toxin [1–6].

Materials

- A <u>NetB-negative</u> *C. perfringens* strain
- TGY broth:
  - 3% tryptone
  - 2% yeast extract
  - 0.1% glucose
  - 0.1% L-cysteine
- 0.2 μm filters
- Vivaspin 20 centrifugal concentrator (5kDa molecular weight cut-off)
- Hanks balanced salt solution (HBSS)
- Quil-A saponin vaccine adjuvant

Procedure

Preparation of the immunogen

<u>Day 1</u>:

- Inoculate a single **<u>NetB-negative</u>** *C. perfringens* colony in 25 ml TGY broth
- Incubate anaerobically at a temperature between 37°C and 42°C, for 16h-24h (overnight)

<u>Day 2:</u>

Concentrate the *C. perfringens* culture supernatants:

- Centrifuge the *C. perfringens* overnight culture (min 4500 x g for 5 min at 4°C)
- Collect the supernatants and filter-sterilize using a 0.2 μm filter
- Add the filtered supernatants to the top compartment of a centrifugal concentrator with a molecular weight cut-off (MWCO) smaller than the MW of *C. perfringens* alpha toxin (~43 kDa) or perfringolysin O (~53 kDa). (e.g. Vivaspin 20 with 5kDa MWCO)
- Centrifuge at 4000 x g for 45 minutes
- Discard the flow-through and wash the supernatants by adding 5 mL HBSS and mix by gently pipetting
- Centrifuge at 4000 x g for 40 minutes
- Discard the flow-through and collect the concentrated supernatants

- Determine the protein concentration using the BCA assay or Bradford method
- Dilute the SN to a concentration of 300µg/mL in HBSS, aliquot and store the SN at -20°C

Rabbit hyperimmunization protocol

- For each rabbit, prepare 300 µl SN [300 µg/ml] + 5µl Quil-A adjuvants [10 mg/ml] (in HBSS)
- Immunize the animals by subcutaneous injection of the antigen preparation.
- A booster immunization should be given at day 14 post the primary immunization.
- Polyclonal serum is collected at 1 to 2 weeks post the booster immunization, depending on the antibody development.

The monitor the antibody development, serum should be collected before the primary immunization (pre-immune serum), before the booster immunization (day 14) and at 1-2 weeks after the booster immunization. Small blood samples to monitor the antibody development is collected from the marginal ear vein using standard techniques.

Antibody development can be measured using ELISA as previously described [6], with *C. perfringens* supernatants or purified toxins (alpha toxin or perfringolysin O) coated on the plates.

Development of alpha toxin and perfringolysin O-neutralizing antibodies in the serum can be monitored using previously published *in vitro* neutralization tests [6].

References:

1. Ito, A. Alpha toxoid of Clostridium perfringens. I. Purification and toxoiding of alpha toxin of C. perfringens. *Jpn J Med Sci Biol* **1968**, *21*, 379–391.
2. Maclennan, J.D. The histotoxic clostridial infections of man. *Bacteriol Rev* **1962**, *26*, 177–276.
3. Titball, R.W. Gas gangrene: an open and closed case. *Microbiology* **2005**, *151*, 2821–2828, doi:10.1099/mic.0.28248-0.
4. Kulkarni, R.R.; Parreira, V.R.; Sharif, S.; Prescott, J.F. Immunization of broiler chickens against Clostridium perfringens-induced necrotic enteritis. *Clin Vaccine Immunol* **2007**, *14*, 1070–1077, doi:10.1128/CVI.00162-07.
5. Titball, R.W. Clostridium perfringens vaccines. *Vaccine* **2009**, *27 Suppl 4*, D44-7, doi:10.1016/j.vaccine.2009.07.047.
6. Goossens, E.; Verherstraeten, S.; Valgaeren, B.R.; Pardon, B.; Timbermont, L.; Schauvliege, S.; Rodrigo-Mocholi, D.; Haesebrouck, F.; Ducatelle, R.; Deprez, P.R.; et al. Toxin-neutralizing antibodies protect against Clostridium perfringens-induced necrosis in an intestinal loop model for bovine necrohemorrhagic enteritis. *BMC Vet Res* **2016**, *12*, 101, doi:10.1186/s12917-016-0730-8.
