# Supplementary material for: A Rapid and Simple Assay Correlates In Vitro NetB Activity with Clostridium perfringens Pathogenicity in Chickens
Source: Microorganisms. 2021 Aug 11;9(8):1708. doi: 10.3390/microorganisms9081708 (PMC8400579; doi:10.3390/microorganisms9081708)
Supplement: Supplementary file 1 [file microorganisms-09-01708-s001.zip › Document S2_Determining minimal serum concentration.pdf]

Determining the optimal antiserum concentration to block non-NetB *C. perfringens* haemolysins

Reagents

Diluent:

Either PBS (phosphate buffered saline) or HBSS (Hanks balanced salt solution without phenol red) can be used as diluent.

2 % chicken red blood cells:

- 10 ml chicken blood (collected from broilers, on EDTA-tubes)
- Centrifuge 1500 x g, 10 min, RT
- Remove supernatant, replace with 10 ml HBSS and resuspend gently
- Centrifuge 1500 x g, 10 min, RT
- Remove supernatant, replace with 10 ml HBSS and resuspend gently
- Centrifuge 1500 x g, 10 min, RT
- Remove supernatant and gently swirl red blood cells
- Take out 1 ml of packed red blood cells and add to 49 ml HBBS (test blood) or 49 ml dH_2O (positive control)
- Place blood at 37°C for 15 min before use
- Mix blood thoroughly before adding to wells

TGY broth:

- 3% tryptone
- 2% yeast extract
- 0.1% glucose
- 0.1% L-cysteine

Procedure

Collection of *C. perfringens* culture supernatants

The haemolytic effect of non-NetB haemolysins produced by *C. perfringens* towards chicken red blood cells, is observed through determining the haemolysis induced by NetB-negative strains. In order to determine the antiserum concentration to block non-NetB haemolysins, it is advised to use at least 4 different NetB-negative *C. perfringens* strains. No effect of the antiserum on the haemolysis induced by NetB-positive strains is expected.

Day 1:

- For each (NetB-negative) strain, inoculate a single *C. perfringens* colony in TGY broth
- Incubate anaerobically at a temperature between 37°C and 42°C, for 16h-24h (overnight)

Day 2:

- Spin down the *C. perfringens* culture at maximum speed for 5 min at 4°C
- Collect the supernatants and filter sterilize using an 0.2 μm filter
- Aliquot the SN and store at -20°C

Repeat the procedure to obtain 3 independently derived supernatants (3 biological replicates) from each strain.

Determine the optimal antiserum concentration

- Set up 96-well plates in duplicate for each supernatant (technical replicates)
- Dilute the *C. perfringens* culture supernatants 1/5 in HBSS (20% supernatants)

Pre-incubation with serum

- Add 100 μl HBSS to all wells except the first well of each row
- Add 160 μl HBSS to the first well of row A to G, but not in row H. (Row H is for positive control blood)
- Add 40 μl antiserum to the first well of row A to G. (Resulting in 200 μl [20% antiserum] in the first row)
- Serially dilute the antiserum 2-fold across the plate with 100 μl volumes until well 11. (Well 12 is the blank)
- Add 100 μl of 20% *C. perfringens* culture supernatants to each well (10% final culture supernatants concentration). One row per *C. perfringens* strain (on duplicate plates).
- Incubate covered plates at 37°C for 30 minutes.
- Prewarm blood at 37°C for 15 minutes

Haemolysis test

- Add 100 μl of 2% RBC in HBSS to each well, except row H (Resulting in 200 μl total volume, with 1% RBC per well)
- To row H, add 100 μl 2% RBC in dH_2O (positive control)
- Incubate covered plates at 37°C for 30 minutes.
- Allow plates to cool to RT
- Spin microtitre plates for 1 min at 1000 x g
- Remove 150 μl of spun down supernatant from each well and transfer to corresponding well in a new microtitre plate. Be careful to not disturb the pelleted red blood cells.
- Read plate at OD = 550 nm
- The minimal antiserum dilution is the last dilution that shows complete inhibition of haemolysis induced by the NetB-negative strains (no difference with blank OD).
- The final antiserum dilution used in the NetB assay is 5x the antiserum dilution that showed complete inhibition of non-NetB haemolysis.

Dilute antiserum 2-fold across plate →

	1	2	3	4	5	6	7	8	9	10	11	12
A	SN1											blank
B	SN2											blank
C	SN3											blank
D	SN4											blank
E	SN5											blank
F	SN6											blank
G	SN7											blank
H	+ve	+ve	+ve	+ve	+ve	+ve	+ve	+ve	+ve	+ve	+ve	blank

SN = supernatants
+ve = positive control: no SN, RBCs in H_2O (100% haemolysis)
Blank = negative control: no SN, RBCs in HBSS (0% haemolysis)
