# Supplementary material for: A Rapid and Simple Assay Correlates In Vitro NetB Activity with Clostridium perfringens Pathogenicity in Chickens
Source: Microorganisms. 2021 Aug 11;9(8):1708. doi: 10.3390/microorganisms9081708 (PMC8400579; doi:10.3390/microorganisms9081708)
Supplement: Supplementary file 1 [file microorganisms-09-01708-s001.zip › Document S3_NetB_doubling dilution assay.pdf]

NetB doubling dilution assay (haemolysis of chicken red blood cells)

Reagents

Diluent:

Either PBS (phosphate buffered saline) or HBSS (Hanks balanced salt solution without phenol red) can be used as diluent.

2 % chicken red blood cells:

- 10 ml chicken blood (collected from broilers, on EDTA-tubes)
- Centrifuge 1500 x g, 10 min, RT
- Remove supernatant, replace with 10 ml HBSS and resuspend gently
- Centrifuge 1500 x g, 10 min, RT
- Remove supernatant, replace with 10 ml HBSS and resuspend gently
- Centrifuge 1500 x g, 10 min, RT
- Remove supernatant and gently swirl red blood cells
- Take out 1 ml of packed red blood cells and add to 49 ml HBBS (test blood) or 49 ml dH_2O (positive control)
- Place blood at 37°C for 15 min before use
- Mix blood thoroughly before adding to wells

TGY broth:

- 3% tryptone
- 2% yeast extract
- 0.1% glucose
- 0.1% L-cysteine

Procedure

Collection of *C. perfringens* culture supernatants

Day 1:

- For each (NetB-positive) strain, inoculate a single *C. perfringens* colony in TGY broth
- Incubate anaerobically at a temperature between 37°C and 42°C, for 16h-24h (overnight)

Day 2:

- Spin down the *C. perfringens* culture at maximum speed for 5 min at 4°C
- Collect the supernatants and filter sterilize using an 0.2 µm filter
- Aliquot the SN and store at -20°C

Repeat the procedure to obtain 3 independently derived supernatants (3 biological replicates) from each strain.

NetB doubling dilution assay (96-well plate format)

- Set up 96-well plates in duplicate for each supernatant (technical replicates)

Pre-incubation with serum

- Add 100 µl HBSS to all wells except the first well of each row
- Add 160 µl HBSS to the first well of row A to G, but not in row H. (Row H is for positive control blood)
- Add 40 µl culture supernatants to the first well of row A to G. (Resulting in 200 µl [20% supernatants] in the first row)
- Serially dilute 2-fold across the plate with 100 µl volumes until well 11. (Well 12 is the blank)
- Add 100 µl of 1% anti-alpha toxin and perfringolysin O serum to each well (0.5% final serum concentration). The optimal dilution for the antiserum is 5x the minimal concentration needed to block the non-NetB haemolytic activity (See supplementary file S2 to determine the correct antiserum dilution).
- Incubate covered plates at 37°C for 30 minutes.
- Prewarm blood at 37°C for 15 minutes

Haemolysis test

- Add 100 µl of 2% RBC in HBSS to each well, except row H (Resulting in 200 µl total volume, with 1% RBC per well)
- To row H, add 100 µl 2% RBC in dH_2O (positive control)
- Incubate covered plates at 37°C for 30 minutes.
- Allow plates to cool to RT
- Spin microtitre plates for 1 min at 1000 x g
- Remove 150 µl of spun down supernatant from each well and transfer to corresponding well in a new microtitre plate. Be careful to not disturb the pelleted red blood cells.
- Read plate at OD = 550 nm
- The NetB titre is obtained by fitting a Hill curve to the concentration-response data and calculating the LD50 value for each strain (supernatant dilution that causes 50% haemolysis).

Dilute supernatants 2-fold across plate →

	1	2	3	4	5	6	7	8	9	10	11	12
A	SN1											blank
B	SN2											blank
C	SN3											blank
D	SN4											blank
E	SN5											blank
F	SN6											blank
G	SN7											blank
H	+ve	+ve	+ve	+ve	+ve	+ve	+ve	+ve	+ve	+ve	+ve	blank

SN = supernatants
+ve = positive control: no SN, RBCs in H_2O (100% haemolysis)
Blank = negative control: no SN, RBCs in HBSS (0% haemolysis)
