# Supplementary material for: A Rapid and Simple Assay Correlates In Vitro NetB Activity with Clostridium perfringens Pathogenicity in Chickens
Source: Microorganisms. 2021 Aug 11;9(8):1708. doi: 10.3390/microorganisms9081708 (PMC8400579; doi:10.3390/microorganisms9081708)
Supplement: Supplementary file 1 [file microorganisms-09-01708-s001.zip › supplementary figures.pdf]

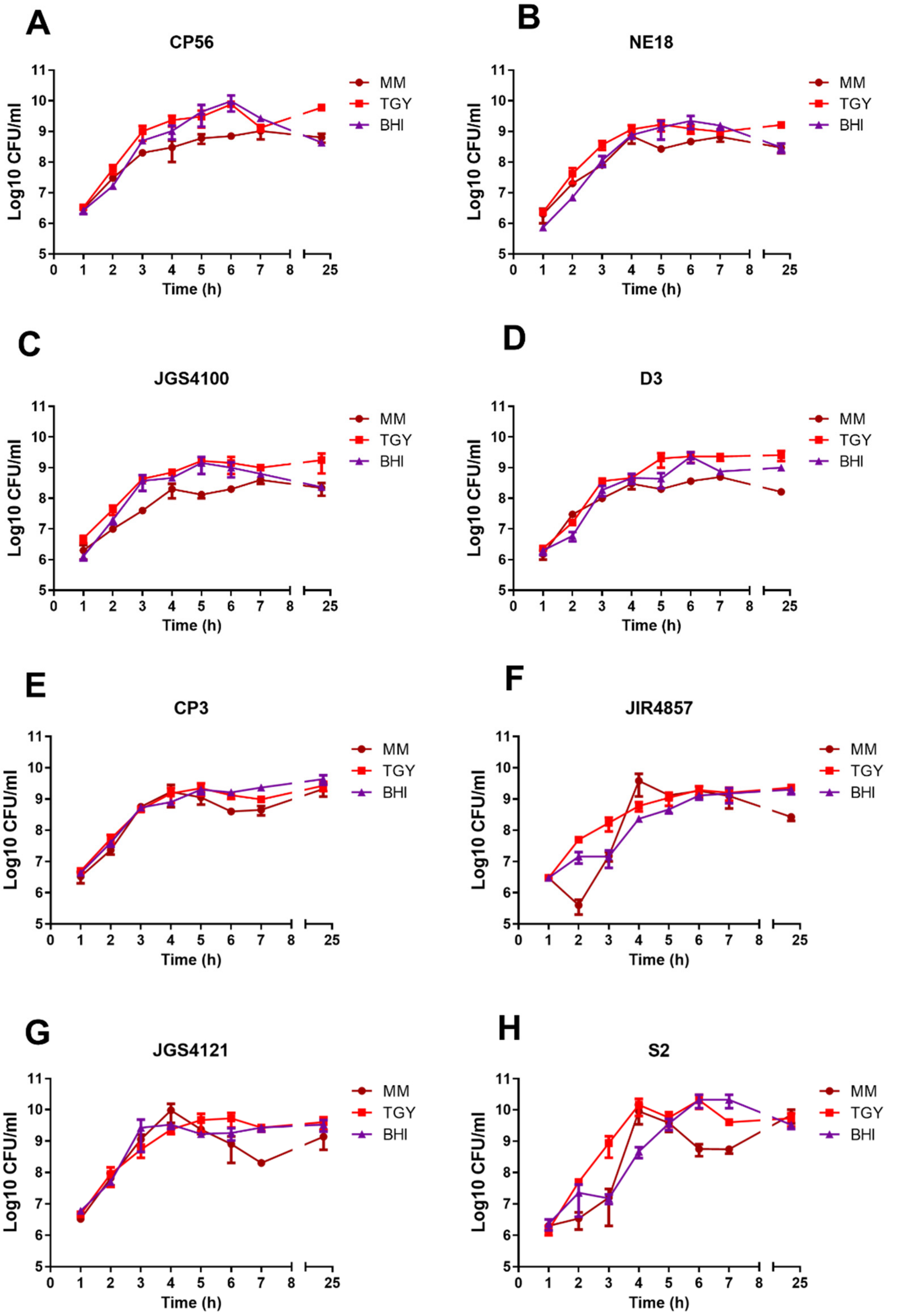

Figure S1. Growth curves of four NetB-positive and four NetB-negative strains in MM, TGY and BHI medium. Each graph shows the growth curves of one strain in MM, TGY and BHI medium. Graphs (A)-(D) show the NetB-positive strains CP56, EHE-NE18, JGS4100 and D3, respectively. Graphs (E)-(H) show the NetB negative strains CP3, JIR4857, JGS4121 and S2, respectively. The x-axis represents the incubation time in hours while the y-axis shows the colony forming units per timepoint (log10 CFU/mL).

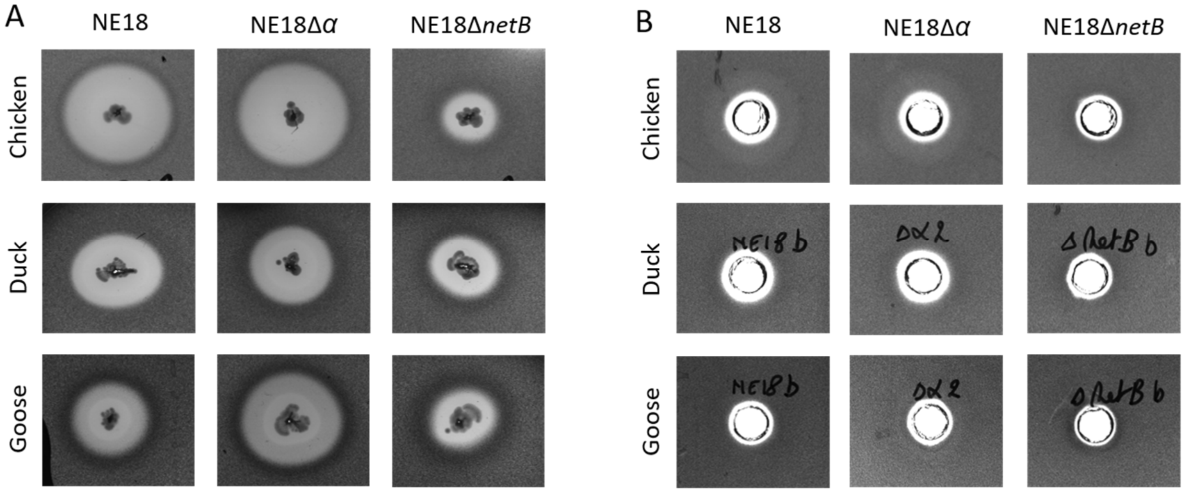

Figure S2. Haemolysis induced by the NetB-positive *C. perfringens* strain NE18, its alpha toxin mutant (NE18 Δα) or its NetB toxin mutant (NE18 Δ*netB*) on Columbia agar supplemented with 5% chicken blood (top), duck blood (middle) or goose blood (bottom row). (A) The strains are directly grown on the avian blood plates. On chicken and duck blood agar plates, the NE18 wild type strain and the alpha toxin mutant strain show an incomplete outer haemolysis zone which is difficult to distinguish from the incomplete inner haemolysis zone. On goose blood agar, the incomplete outer and inner haemolysis zone are easier to distinguish. On none of the avian blood agar plates the NE18 NetB toxin mutant strain (NE18 Δ*netB*) did produce an outer haemolysis zone. (B) The strains were grown overnight in TGY broth, after which the SN was collected and added to wells in the avian blood agar plates. On all avian blood types, complete haemolysis was induced by the culture supernatants from all strains. Only on chicken blood agar, an additional faint outer haemolysis zone could be observed for the culture supernatants of strains NE18 and its alpha toxin mutant (NE18 Δα). No outer haemolysis zone was observed for the culture supernatants of the NetB mutant (NE18 Δ*netB*) on chicken blood. On duck and goose blood no outer haemolysis zone was induced by the culture supernatants.
